# Total energy of sigma-phase Fe–Cr–X (X=Co, Ni) alloys: Calculated and modeled data

**DOI:** 10.1016/j.dib.2016.11.064

**Published:** 2016-12-09

**Authors:** J. Cieślak, J. Tobola

**Affiliations:** AGH University of Science and Technology, Faculty of Physics and Applied Computer Science, PL-30-059 Krakow, Poland

## Abstract

The article contains computational data of electronic structure and crystal stability of two sigma-phases, namely Fe–Cr–Co and Fe–Cr–Ni, using the Korringa–Kohn–Rostoker method (KKR) for electronic band structure calculations. Total energy values, *E*_*T*_, calculated for the number of ordered unit cells with various atomic concentrations and sublattice occupancies are reported. In parallel, obtained data are modelled assuming polynomial dependence of the *E*_*T*_-values versus sublattice occupancies. For more details, please see the article “Site occupancies in sigma-phase Fe–Cr–X (X=Co, Ni) alloys: Calculations versus experiment” (J. Cieslak, J. Tobola, S.M. Dubiel, 2016) [1].

**Specifications Table**

**Table:** 

Subject area	*Physics*
More specific subject area	*Condensed matter physics, Materials science*
Type of data	*Tables*
How data was acquired	*Electronic structure calculations by the KKR and KKR-CPA methods*
Data format	*Calculated, modeled and fitted*
Experimental factors	*Fe–Cr–X (X=Ni, Co) sigma phase samples, as cast*
Experimental features	*RT standard measurements, DFT calculations using KKR technique*
Data source location	*AGH University of Science and Technology, Faculty of Physics and Applied Computer Science, PL-30–059 Krakow, Poland*
Data accessibility	*Data is with article*

**Value of the data**

• One can continue the calculations using the same format of the data.
• One can compare the data with other theoretical results.
• One can verify the validity of other models and approaches.
• One can predict site occupancies for other alloy contents.

## 1 Data

The assumed input parameters and the results of calculations and modelling are shown in Tables. They contain atomic positions used for calculations (common for both sigma-Fe–Cr–Co and sigma-Fe–Cr–Ni systems), atomic occupancies of the above-defined atomic positions used for calculations as well as total energy values calculated for such-defined unit cells. Polynomial coefficients determined from the fit to the KKR calculation results are presented also.

## 2 Experimental design, materials and methods

### 2.1 KKR electronic structure calculations: Input parameters

For all computations atomic positions in the sigma-phase unit cell were the same, as indicated in Table 1. They were obtained from neutron diffraction measurements. Specific atomic occupancies of the unit cell for which calculations were performed are given in Tables 2a and 2b. They were chosen randomly, covering the whole range of the sigma-phase existence in discussed systems.

#### 2.1.1 KKR electronic structure calculations: results

Total energy values, *E*_*T*_, calculated for the number of ordered unit cells as well as corresponding values indicated from the polynomial fit are given in Tables 3a and 3b. Please remember, that *E*_*T*_ value corresponds to the whole unit cell (30 atoms in this case) with specific atomic arrangement, but not to particular atoms. Such values can be calculated by fitting *E*_*T*_ –values and corrections originating from particular nearest neighbor numbers, or, in other words, sublattice occupancies. Obtained polynomial coefficients are shown in Tables 4a and 4b. All calculation details are given in the paper [1].

## Footnotes

Appendix A Supplementary material associated with this article can be found in the online version at doi:10.1016/j.dib.2016.11.064.
